# Tussilagone mitigates sepsis-induced acute lung injury in mice by suppressing *RIPK1* expression

**DOI:** 10.1186/s41065-026-00678-7

**Published:** 2026-04-21

**Authors:** Jie Gu, Yachen Cai, Jiangwen Yin, Mengjie Zhang, Jun Chen, Hongjun Miao

**Affiliations:** 1 Department of Emergency/Critical Care Medicine, Children’s Hospital of Nanjing Medical University, No. 72 GuangZhou Road, Nanjing, Jiangsu Province 210008 People’s Republic of China; 2 Department of Emergency Medicine, Children’s Hospital of Soochow University, Suzhou, Jiangsu Province 215000 People’s Republic of China

**Keywords:** ALI, Network pharmacology, *RIPK1*, Sepsis, Tussilagone

## Abstract

**Objective:**

Sepsis is a severe condition characterized by life-threatening organ dysfunction resulting from an excessive host response to infection. Among the affected organs, the lungs are particularly susceptible during the progression of sepsis. Tussilagone (TS), a sesquiterpenoid compound derived from the flowers of *Tussilago farfara*, has been explored for its potential therapeutic effects on sepsis-induced lung injury. This study used both in vitro and in vivo experimental approaches, complemented by network pharmacology analyses, to verify the mechanisms and molecular targets underlying the effects of TS.

**Methods:**

TS was administered to mice with sepsis-induced lung injury and to lung epithelial cells stimulated with lipopolysaccharide (LPS) to assess its therapeutic potential. Inflammatory factor levels in mouse serum, lung tissue, and cells were assessed. Western blot analysis was used to determine the expression of NF-κB p65 and MyD88 in lung tissues. Potential molecular TS targets were identified, with receptor-interacting serine/threonine-protein kinase 1 (*RIPK1*) selected for further investigation using the Traditional Chinese Medicine Systems Pharmacology Database and Analysis Platform. Additionally, the study explored if TS exerts its protective effects against lung injury in septic mice through targeted regulation of *RIPK1*.

**Results:**

TS inhibited the NF-κB p65 and MyD88 signaling pathways, reducing the release of inflammatory factors and a mitigation of lung injury severity in LPS-induced MLE12 cells and CLP-induced septic mice. Overexpression of RIPK1 protein resulted in elevated expression levels of NF-κB p65 and MyD88, whereas treatment with TS significantly reduced these levels. Similarly, the use of a RIPK1 protein inhibitor to suppress *RIPK1* expression also decreased the expression of NF-κB p65 and MyD88, further supporting the role of *RIPK1* in the mechanism of TS-mediated lung protection.

**Conclusion:**

TS demonstrates a protective effect against sepsis-induced acute lung injury (ALI) by inhibiting the expression of *RIPK1*. These findings indicate that TS can potentially be a therapeutic agent for the treatment of ALI.

## Introduction

Sepsis is defined as life-threatening organ dysfunction resulting from a dysregulated host response to infection [1]. It represents a severe and uncontrolled reaction to infection, leading to tissue damage, organ failure, and potentially death [2]. Globally, approximately 31.5 million cases of sepsis and 19.4 million cases of severe sepsis occur annually, contributing to an estimated 5.3 million deaths [3]. A data-analysis of 27 studies conducted in 7 high-income countries revealed an incidence of 437 cases of sepsis and 270 cases of severe sepsis per 100,000 person-years over the past decade [4]. During this period, the in-hospital mortality rates for sepsis and severe sepsis were reported as 17% and 26%, respectively, with septic shock accounting for a case fatality rate as high as 50%. This high mortality rate makes sepsis a significant contributor to fatalities among patients in the intensive care unit [5]. 

Despite advances in understanding the pathophysiology, clinical characteristics, and management strategies for sepsis, the underlying mechanisms driving its progression are not completely understood [6]. Effective and specific treatment options are limited, and the mortality rate remains alarmingly high, ranging from 30% to 70% [7]. Moreover, the economic burden of sepsis is substantial due to high treatment costs and significant consumption of medical resources, posing a serious challenge to public health and safety.

Addressing the complexities of sepsis requires a comprehensive understanding of its pathogenesis, active exploration of novel therapeutic approaches, and further elucidation of the mechanisms underlying its onset and progression. Developing effective intervention strategies holds the potential to revolutionize sepsis treatment, improving patient outcomes, reducing mortality rates, and conserving medical resources.

The lungs are the organs most commonly affected during the onset and progression of sepsis. Sepsis-induced lung injury is characterized by the activation of inflammatory signaling pathways, macrophage activation, and the subsequent release of large quantities of inflammatory mediators. This results in extensive inflammatory infiltration within the lungs, severe damage to capillary endothelial and epithelial cells, and clinical manifestations such as diffuse pulmonary inflammatory infiltrates, pulmonary edema, respiratory distress, and refractory hypoxemia. These complications significantly threaten patient survival [8]. 

The core of sepsis-induced lung injury lies in aspects such as inflammatory activation and cellular damage and effective therapeutic strategies are currently lacking. Therefore, further investigation into the pathogenesis of sepsis-related lung injury and the development of effective pharmacological interventions hold promise for reducing patient mortality and improving long-term quality of life.

Various treatments have been developed to reduce the mortality associated with sepsis. However, findings from over 30 clinical trials evaluating a range of anti-cytokine and anti-inflammatory drugs have demonstrated limited efficacy, with some studies reporting reduced survival rates. These results underscore the challenges posed by the complexity of the sepsis pathophysiological system and the limitations of current therapeutic approaches [9]. 

Consequently, identifying novel therapeutic agents and targets remains crucial to improving survival rates, particularly in cases of sepsis complicated by organ dysfunction. In recent years, there has been an increasing focus on proprietary Chinese medicines, individual herbal medicines, and their active compounds as potential therapeutic agents for sepsis. These interventions have indicated growing promise in modulating inflammatory responses associated with sepsis, leading to reductions in inflammatory markers and patient mortality rates. Their unique therapeutic properties, combined with minimal side effects, have garnered significant attention from both researchers and clinicians [10]. 

Tussilagone (TS), a sesquiterpenoid compound derived from the flower of *Tussilago farfara*, has been recognized in traditional Chinese medicine (TCM) for its therapeutic properties [11]. Recent research highlights its anti-inflammatory, anticancer, and antioxidant activities, supporting its use in the treatment of asthma, bronchitis, diabetic obesity, cancer, and various inflammatory conditions [12, 13]. Sepsis is attributed to a deficiency of vital qi, internal accumulation of pathogenic factors, and disturbances in blood and qi circulation, ultimately leading to impaired visceral function, based on TCM principles [10]. Blood stasis is considered a key pathogenic factor in this context.

TS alleviates sepsis-induced lung injury through multiple mechanisms, including promoting blood circulation, resolving stasis, alleviating cough, resolving phlegm, and exerting anti-inflammatory effects [14]. Mechanistically, TS inhibits the production of inflammatory mediators in lipopolysaccharide-stimulated monocyte-macrophages via the NF-κB and p38 mitogen-activated protein kinase (MAPK) signaling cascades [15]. Additionally, TS regulates cancer cell proliferation, angiogenesis, and inflammatory responses through pathways involving Wnt, VEGFR2, IRF, and heme oxygenase-1 [16]. It also reduces the production of inflammatory cytokines, such as IL-1β and tumor necrosis factor-α (TNF-α), by targeting Myeloid Differentiation Primary Response Protein 88 (MyD88)- and TRIF-dependent pathways downstream of TOLL Like receptor 4 (TLR4) in dendritic cells [17]. 

Despite these findings, the specific role and mechanisms of TS in the pathological processes of sepsis-induced lung injury, particularly in children and teenagers, remain to be fully elucidated. Further research is necessary to clarify its therapeutic potential in this context.

Receptor-interacting serine/threonine-protein kinase 1 (*RIPK1)* is a key member of the receptor interacting protein (RIP) kinase family [18]. It plays a key role in regulating various signaling pathways that influence cellular fate, particularly in apoptosis and programmed necrosis, where its kinase activity is pivotal [19]. However, the potential of RIPK1 as a therapeutic target of TS in the treatment of sepsis-related lung injury has not been reported. In this study, we employed a network pharmacology approach—a bioinformatics prediction based on functional genomics for the core targets of TS.

## Materials and methods

### Prediction and verification of TS theoretical targets

#### TS target prediction

The chemical constituents and potential targets of TS were identified using the Traditional Chinese Medicine Systems Pharmacology Database and Analysis Platform (TCMSP) [20]. The target-associated genes were retrieved from the UniProt database and subsequently analyzed using the STRING database (https://string-db.org/) to determine protein-protein interaction networks. The anti-inflammatory mechanisms of TS were further predicted through WebGestalt Gene Ontology (GO) analysis and Kyoto Encyclopedia of Genes and Genomes (KEGG) pathway analysis.

A total of 243 KEGG pathways were identified as significantly associated *(p ≤* 0. 05). Among the top anti-inflammatory-related pathways were the MyD88 signaling pathway, MAPK signaling pathway, and TLR4 signaling pathway. The target genes involved included *RIPK1*, *MAPKAPK2*, *MAPK9*, *MAPK11*, *MAP2K1*, *CDK1*, *RPS6KA3*, *TBK1*, and others, ranked by their degree of association.

#### Molecular docking technology validation

The 3D protein structure file in Protein Data Bank (PDB) format was obtained from the Research Collaboratory for Structural Bioinformatics (RCSB) PDB (https://www.rcsb.org/). Ligand and water molecules were removed from the PTGS2 protein structure, and the modified file was saved in PDB format using Discovery Studio 4.5 Client software. The two-dimensional structure file of the active compound, in Structure Data File format, was downloaded from the PubChem database (https://pubchem.ncbi.nlm.nih.gov/).

PyRx software was used for molecular docking. Initially, the receptor files were dehydrogenated and converted into Protein Data Bank, Partial Charge (Q), & Atom Type (T) (PDBQT) format. Subsequently, the ligand compound files were subjected to energy minimization and converted into PDBQT format. AutoDock Vina was used for molecular docking. A binding energy of less than 0 indicated that the ligand could spontaneously bind to the receptor. If the binding energy mode and the RMSD lower/upper bounds were 0, the conformation with the lowest binding energy was selected, indicating a stable conformation with a higher likelihood of interaction.

The docking results were analyzed and visualized using Discovery Studio 4.5 Client software, and the output was saved as PNG files.

### Cecal ligation and perforation (CLP)-induced septic lung injury mouse model and the treatment of TS

Specific Pathogen Free (SPF)-grade male C57BL/6 mice (aged 4–6 weeks, weighing 18–20 g) were purchased from Nanjing Viatroni Experimental Animal Technology Co., Ltd. All animals were acclimatized for one week in the specific pathogen-free facility of Nanjing Medical University’s Animal Experiment Center prior to experimentation. The mice were acclimated for one week, and they were fasting and water-free for 6 h before surgery. Pentobarbital sodium was injected intraperitoneally at 50 mg/kg to anesthetize the mice. The anesthetic effect was evaluated by pinching the toes with forceps 5–10 min after anesthesia. Thirty-six mice were randomly divided into three groups: Sham operation group (Sham group, *n* = 10), sepsis group (CLP group, *n* = 13), and tussilagone intervention group (TS + CLP group, *n* = 13). Mice in the TS + CLP group were intragastrically administered with 30 mg/kg TS 1 h before CLP, while the Sham group and CLP group were given an equal volume of normal saline. In the CLP [21] group, the cecum was isolated and ligated at the midpoint of the distal cecum, ensuring the ileocecal valve remained unaffected. The cecal contents were gently displaced toward the distal end, and the cecum was perforated 0.5 cm from the blind end using a 21-gauge needle. A small amount of cecal contents was expressed using forceps. Following the surgical procedure, each group received a subcutaneous injection of normal saline. The study [22] found that daily oral administration of 25 mg/kg TS significantly alleviated colon inflammation in mice after 10 days compared to the control group. Therefore, we selected three different TS concentrations—10 mg/kg, 20 mg/kg, and 30 mg/kg—for oral administration before the CLP surgery in mice, followed by Enzyme-Linked Immunosorbent Assay (ELISA) analysis of serum inflammatory factors. The results indicated that 30 mg/kg TS was the most effective dose. TS (Shanghai Jinshui Biotechnology Co., LTD. China) at a dose of 30 mg/kg or an equivalent volume of dimethyl sulfoxide (DMSO) (D2650, Sigma, USA) was administered by gavage to the CLP model mice one hour prior to the procedure.

After anesthesia, the mice were sacrificed by cervical dislocation. Blood samples were clotted at room temperature for 30–40 min, then centrifuged at 1,000 × g (15 min, 4 °C). Serum was collected in 1.5 mL tubes for immediate analysis or stored at -20 °C (< 72 h), following the ELISA kit manufacturer’s instructions. And histological analysis of lung tissue was conducted using hematoxylin and eosin (HE) staining to observe pathological morphological changes. Immunofluorescence techniques and immunoblotting of proteins related to inflammatory pathways in lung tissue were conducted to assess the effects of the intervention on lung injury.

### In vitro lipopolysaccharide (LPS)-stimulated cell model and the treatment of TS

Mouse lung epithelial cells (MLE12) (iCell/Sabak, China) were used as the experimental model, and a sepsis cell model was established by culturing the cells in Dulbecco’s Modified Eagle’s medium/F12 medium (C11330500BT, GIBCO, USA) supplemented with 10% fetal bovine serum (26010074,GIBCO, USA), followed by stimulation with LPS (L2880, Sigma, USA) at a concentration of 10 µg/mL for 8 h. To examine the effects of TS on LPS-stimulated MLE12 cells, TS was applied as a pretreatment for 1 h before LPS stimulation (10 µg/mL) for an additional 8 h.

Subsequently, the samples were collected and centrifuged at 1,000 × g for 10 min (4℃). The supernatant was used to measure inflammatory factors using ELISA. The cells were washed three times with phosphate buffer saline (PBS), and cell lysates (P0013b, Shanghai Biyuntian Biotechnology Co., LTD. China) or TRIzol lysates (15596026, Invitrogen, USA) were added to assess changes in intracellular protein levels and mRNA expression.

### Western-blot

#### Protein extraction from mouse lung tissue

To prepare the cracking fluid, a mixture of PMSF and RIPA at a ratio of 100:1 was prepared. Frozen lung tissues were retrieved and thawed in a refrigerator set at 4 ℃. A 25 mg tissue sample was weighed for each specimen, placed into a 1.5 mL eppendorf (EP) tube, and cut into smaller pieces. Two pre-cooled magnetic beads were added to the tube along with 1 mL of lysis buffer. The tissue was homogenized in a pre-cooled homogenizer for 5–10 min without being disrupted manually.

The supernatant containing the protein was collected, and 10 to 20 µL was reserved for protein concentration measurement. The remaining protein extract was mixed with 5× SDS loading buffer at a 4:1 ratio and heated at 100 °C for 5 min to denature the proteins, making them suitable for subsequent protein expression analysis.

#### Cell protein extraction

The culture medium was discarded, and the wells were washed three times with PBS. A total of 100 µL of lysis buffer was added to each well of a 6-well plate, and the plate was kept on ice for 15 min. The cells in each well were repeatedly scraped for 3 to 5 min until visible foam was formed, ensuring thorough lysis. The resulting cell lysate was transferred to EP tubes and centrifuged at 12,000 × g for 15 min at 4 ℃.

#### Electrophoresis

Clean and dry glass plates. Assemble them with the short plate facing outward.Gel Preparation and Electrophoresis: Prepare separation gel using the Yaase PAGE Gel Rapid Preparation Kit. Load gel between plates, install in the tank, and check for leakage. Add 1× electrophoresis buffer, remove the comb, load protein samples and marker, and run electrophoresis at 100 V for 90 min until the bromophenol blue dye front is ~ 1 cm from the bottom of the lower gel. Separate the short plate and gel. Activate the PVDF membrane in methanol for 30 s, soak filter paper in 1× transfer buffer. Assemble the transfer “sandwich” (sponge → filter paper → gel → PVDF membrane → filter paper → sponge), secure in the wet box, place in the tank in an ice bath. Transfer at 300 mA for 70 min.Block the PVDF membrane with 5% skim milk, incubate with primary antibody overnight at 4 °C, wash, and then incubate with secondary antibody for 1 h at RT. Mix equal volumes of ECL solution A and B, apply to the membrane, expose in the imaging system. Analyze band gray values with Image Lab, using glyceraldehyde − 3-phosphate dehydrogenase (GAPDH) as the internal reference. Antibodies used are as follows: Nuclear factor kappa-B(NF-κB p65), MyD88 and RIPK1 antibodies were purchased from (Abcam, USA), and RIPK1 antibodies were purchased from (Affinity, USA).

### Transfection

The overexpression RIPK1 plasmid used in this study was constructed by Jinbei Gene.

The 6-hole plate was laid, and the cells were transfected when they grew to the logarithmic phase. The groups (*n* = 3) were divided into: the serum-free Dulbecco’s Modified Eagle Medium (DMEM) medium was changed to culture for 30 min before transfection;

Transfection reagent: 100 µl of transfection reagent containing 1% double antibody and serum-free complete medium diluted 3 times the volume of plasmid mass is required for each well; Each hole required 100 µl of 1% double antibody serum-free DMEM medium diluted with 2ug plasmid and placed in an EP tube; Add the prepared transfection reagent to the EP tube, gently bounce, and leave at room temperature for 15 min; Add 198 µl lipo and plasmid mixture to each hole of the six-hole plate; The cells were incubated in a cell incubator for 4 h, and then transferred to complete culture medium for further culture for 24 h; The complete culture medium containing 2.0 mg/ml puromycin was cultured for 2d, and then the complete culture medium without puromycin was used for culturing. When the cells transfected with plasmid reached 80%, the cells were collected and RNA was extracted; The cell culture holes transfected with Vector were used as the control group, and the cell culture holes transfected with RIPK1 shRNA were used as the experimental group. Quantitative Real-Time PCR (qRT-PCR) was used to detect the expression of RIPK1 mRNA in each group (set duplicate holes) to verify the success of transfection.

### Quantitative real-time PCR

Total RNA was extracted from mouse lung tissues and cells. RNA purity (OD260/280 ratio 1.8–2.0) and concentration were verified via a One Drop analyzer. Genomic DNA was removed by DNase I treatment. CDNA was synthesized from 1 µg RNA using PrimeScript RT Master Mix (TaKaRa) with conditions: 37 °C for 15 min, 85 °C for 5 s.Reactions were performed in triplicate using SYBR Green Premix Ex Taq II (TaKaRa) on a Bio-Rad CFX96 system.Relative expression was calculated by the 2^(-ΔΔCt) method normalized to GAPDH. Technical replicates with SD > 0.5 were excluded. Statistical analysis used Student’s t-test. Primers used were listed as fellow: the TNF-α and primers, sense 5-CAGGCGGTGCCTATGTCTC-3 and antisense 5-CGATCACCCCGAAGTTCAGTAG-3; IL-6primers, sense 5-CTGCAAGAGACTTCCATCCAG-3 and antisense 5-AGTGGTATAGACAGGTCTGTTGG-3.

### Statistical analysis

Data were analyzed and visualized using GraphPad Prism software (version 8.0), Image-Lab, and Image J software. Measurement data are presented as mean ± standard deviation (¯x ± s). A one-way analysis of variance (ANOVA) was employed to compare multiple groups of data, followed by post-hoc tests where appropriate. Differences were considered statistically significant at *P* < 0.05.

## Results

To examine the effect of TS on the inflammatory response associated with CLP-induced sepsis-related lung injury in juvenile mice, C57BL/6 mice were used. TS (30 mg/kg) or an equivalent volume of DMSO was administered via oral gavage to the CLP model mice one hour before modeling. Samples were collected 24 h post-modeling to assess serum inflammatory factor levels. Lung injury was evaluated using HE staining, while the expression of the inflammatory protein MyD88 in lung tissue was analyzed via immunofluorescence. WB was performed to detect the expression levels of MyD88 and NF-κB p65 in lung tissues.

**Fig. 1:**
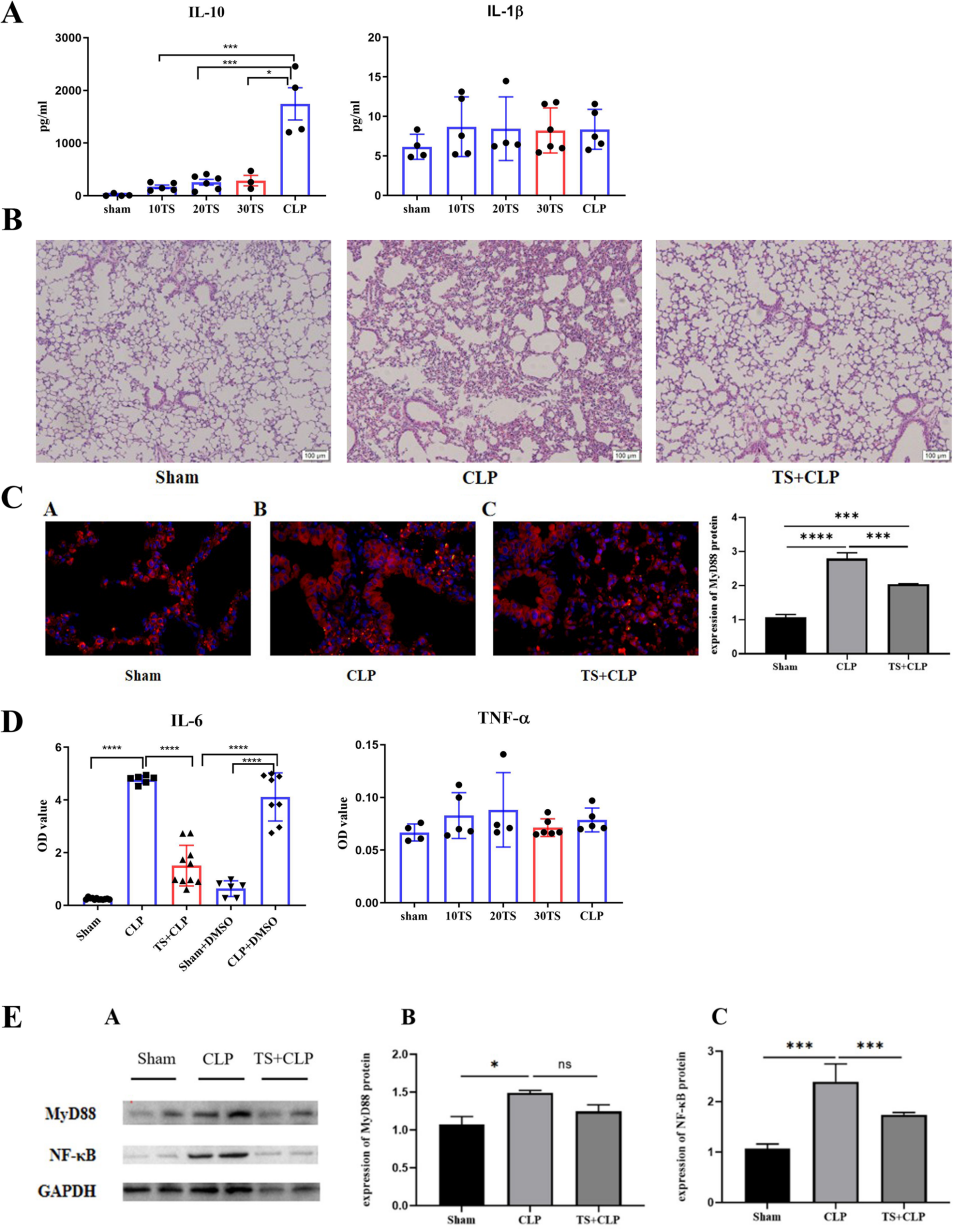
Experimental design illustrating the grouping of 4–5-week-old C57BL/6 mice into Sham, CLP, and TS + CLP groups (15 mice per group). TS (30 mg/kg) or an equivalent volume of DMSO was administered via oral gavage to the CLP model mice one hour before modeling. Samples were collected 24 h post-modeling for analysis. **A** CLP mice treated with 30 mg/kg TS exhibited the highest serum IL-10 concentration and the lowest IL-1β level, suggesting this dosage as the most effective in modulating the inflammatory response. **B** Pathological analysis of lung tissue: HE staining of mouse lung tissue (×400) revealed significant pathological changes in the CLP group compared to the sham group, including alveolar dilation and collapse, widened alveolar septa, extensive vascular congestion, and infiltration of inflammatory cells. These pathological changes were alleviated in the TS intervention group. **C** Immunofluorescence analysis of MyD88 in lung tissue: Immunofluorescence staining indicated blue fluorescence for nuclei and red fluorescence for MyD88. An increased expression of MyD88 was observed in the CLP group compared to the sham group, while TS intervention reduced MyD88 expression; The expression level of MyD88 was significantly elevated in the lung tissues of the CLP group, while it was significantly reduced in the TS + CLP group. **D** Levels of inflammatory cytokines TNF-α and IL-6: ELISA indicated elevated levels of TNF-α and IL-6 in the CLP group. These levels were significantly reduced following TS intervention. **E**. Protein expression of MyD88 and NF-κB p65: Western blot analysis demonstrated elevated protein levels of MyD88 and NF-κB p65 in the CLP group, which were significantly decreased after TS intervention. Statistical analysis revealed significant differences: *P < 0.05, **P < 0.01, ***P < 0.001, ****P < 0.0001

The results indicated that TS intervention significantly reduced lung inflammation in CLP mice compared to the control group. Levels of the inflammatory cytokines TNF-α and IL-6 were significantly decreased, and the expression of inflammatory pathway proteins MyD88 and NF-κB p65 in lung tissue was also reduced (Fig. 1). These findings indicate that TS administration in vivo can alleviate inflammation associated with CLP-induced sepsis-related lung injury.

To further examine the effects of TS on the inflammatory response in LPS-induced mouse lung epithelial cells, MLE12 cells were pretreated with TS for 1 h prior to 8 h of LPS stimulation. Supernatants and cell lysates were collected, and intracellular inflammatory factors were quantified using qRT-PCR. The expression levels of MyD88 and NF-κB p65 were assessed via WB.

**Fig. 2:**
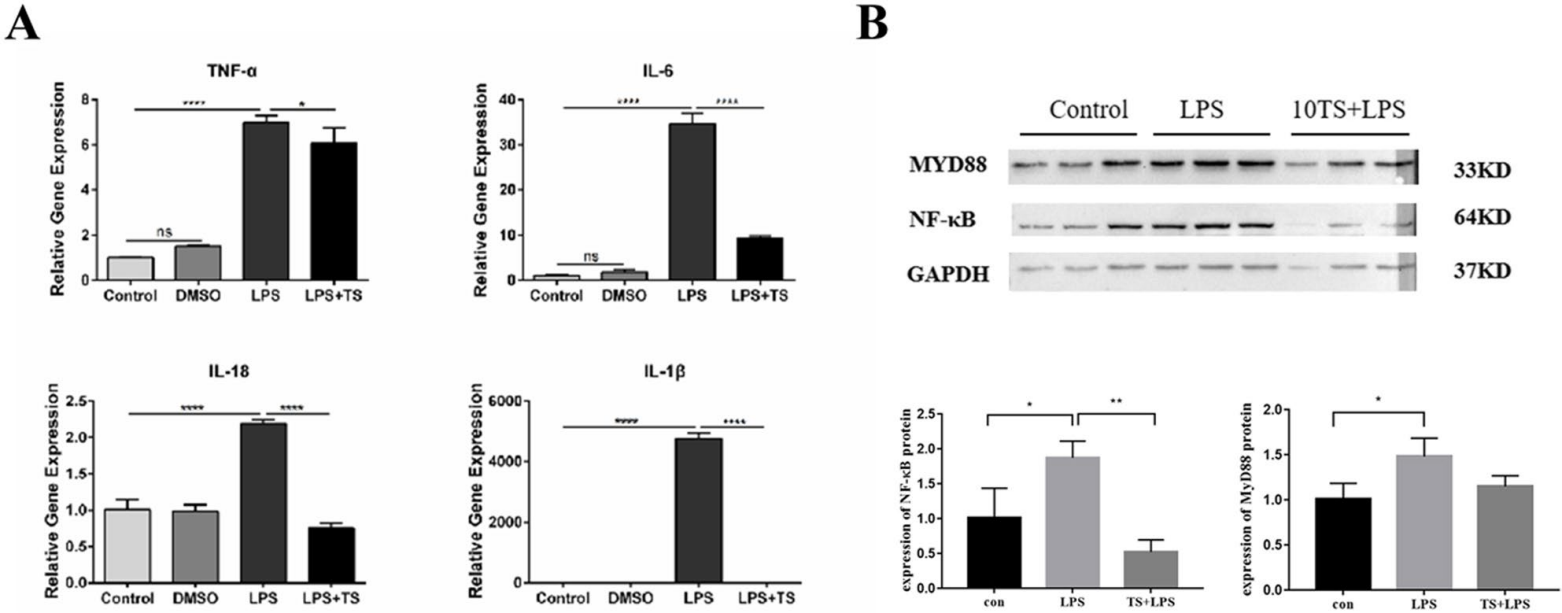
Experimental setup for MLE12 cells treated with TS for 1 h prior to LPS stimulation for 8 h. Supernatants and cells were collected for analysis. **A** Inflammatory cytokine levels: The expression levels of inflammatory cytokines TNF-α, IL-6, IL-1β, and IL-18 were assessed using qRT-PCR after TS intervention. The results indicated a significant reduction in the expression of these inflammatory factors following TS treatment. **B** Protein levels of MyD88 and NF-κB: Western blot analysis was conducted to assess the protein expression levels of MyD88 and NF-κB p65: across the experimental groups. TS intervention resulted in a marked decrease in MyD88 and NF-κB p65: protein levels. Statistical significance was reported as follows: ^*^P < 0.05, ^**^P < 0.01, ^****^P < 0.0001

The results demonstrated that TS pretreatment attenuated the inflammatory response induced by LPS in MLE12 cells (Fig. 2). These findings indicate that TS intervention in vitro can effectively mitigate the LPS-induced inflammatory response in lung epithelial cells.

**Fig. 3:**
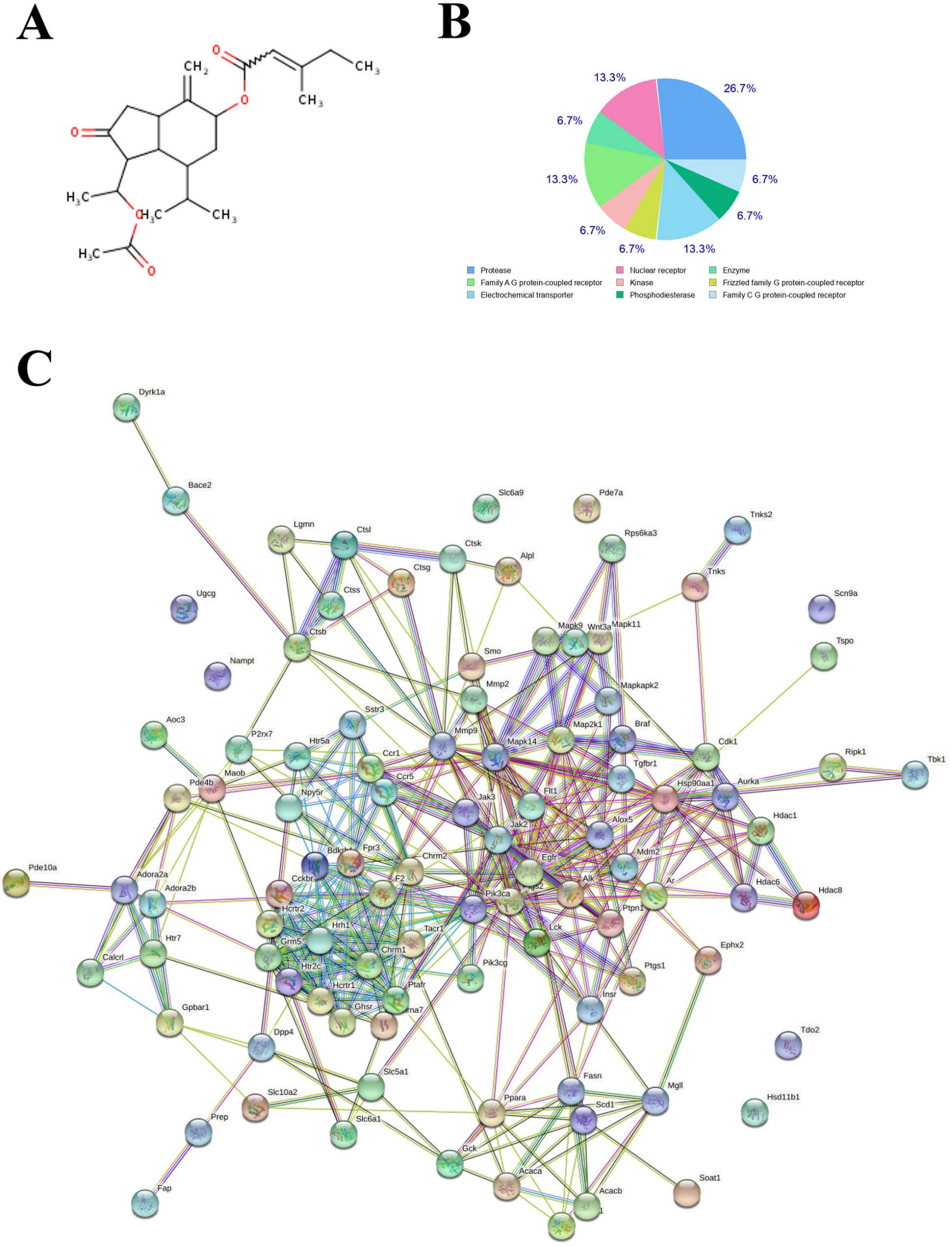
Identification of TS targets using the TCMSP database and analysis platform.The chemical components and potential targets of Tussilagone , a related compound, were also explored. Target-associated genes were retrieved from the UniProt database and analyzed using the STRING protein-protein interaction platform (https://string-db.org/) to determine protein interaction networks. **A**. Molecular formula of Tussilagone: The molecular structure and composition of Tussilagone were characterized, providing insights into its chemical properties. **B-C** Bioinformatics analysis: A bioinformatics approach was used to classify TS targets and map the relationships among 102 target proteins

To further elucidate the specific molecular pathways through which TS alleviates sepsis-related lung injury and identify potential drug targets, the chemical composition and potential targets of TS were analyzed using TCMSP. Target-associated genes were retrieved from the UniProt database and further analyzed using the STRING protein-protein interaction platform (https://string-db.org/) to determine protein interaction networks (Fig. 3).

**Fig. 4:**
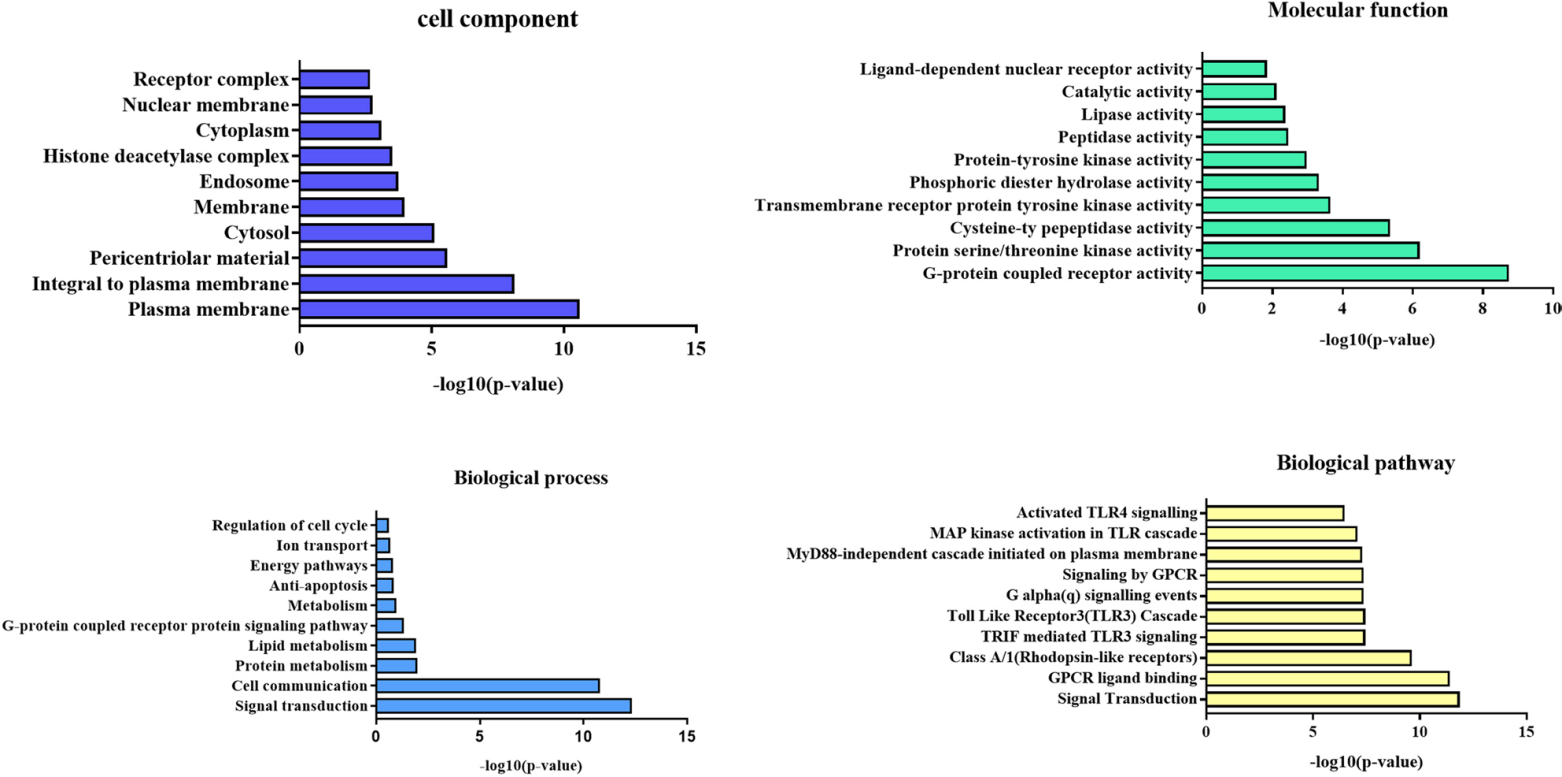
GO and KEGG pathway analyses of theoretical TS target genes, revealing insights into their potential biological roles and mechanisms of action. Biological processes: TS target genes were enriched in processes such as signal transduction, protein metabolism, and other essential cellular activities, indicating their involvement in fundamental regulatory pathways. Cellular components: Target genes were associated with cellular components such as the cell membrane, receptor complexes, and the cytoplasm, highlighting their roles in intracellular and extracellular signaling mechanisms. Molecular functions: Molecular function analysis revealed that TS targets were involved in activities such as protein tyrosine kinase activity and G-protein-coupled receptor activity, reflecting their participation in enzyme-mediated and receptor-driven signaling processes. Pathway analysis: KEGG pathway analysis identified critical pathways associated with TS targets, including signal transduction and the activation of NF-κB p65: and MAP kinases mediated by the TLR4 signaling pathway

Subsequent GO and KEGG pathway analyses were conducted via WebGestalt to predict the anti-inflammatory mechanisms of TS. KEGG pathway enrichment identified 243 signaling pathways significantly associated with TS (*p* ≤ 0.05) (Fig. 4). Among the most relevant anti-inflammatory pathways were the MyD88 signaling pathway, MAPK signaling pathway, and TLR4 signaling pathway. Target genes associated with these pathways included *RIPK1*, *MAPKAPK2*, *MAPK9*, *MAPK11*, *MAP2K*1, *CDK1*, *RPS6KA3*, *TBK1*, and others, ranked by interaction degree. 

**Fig. 5:**
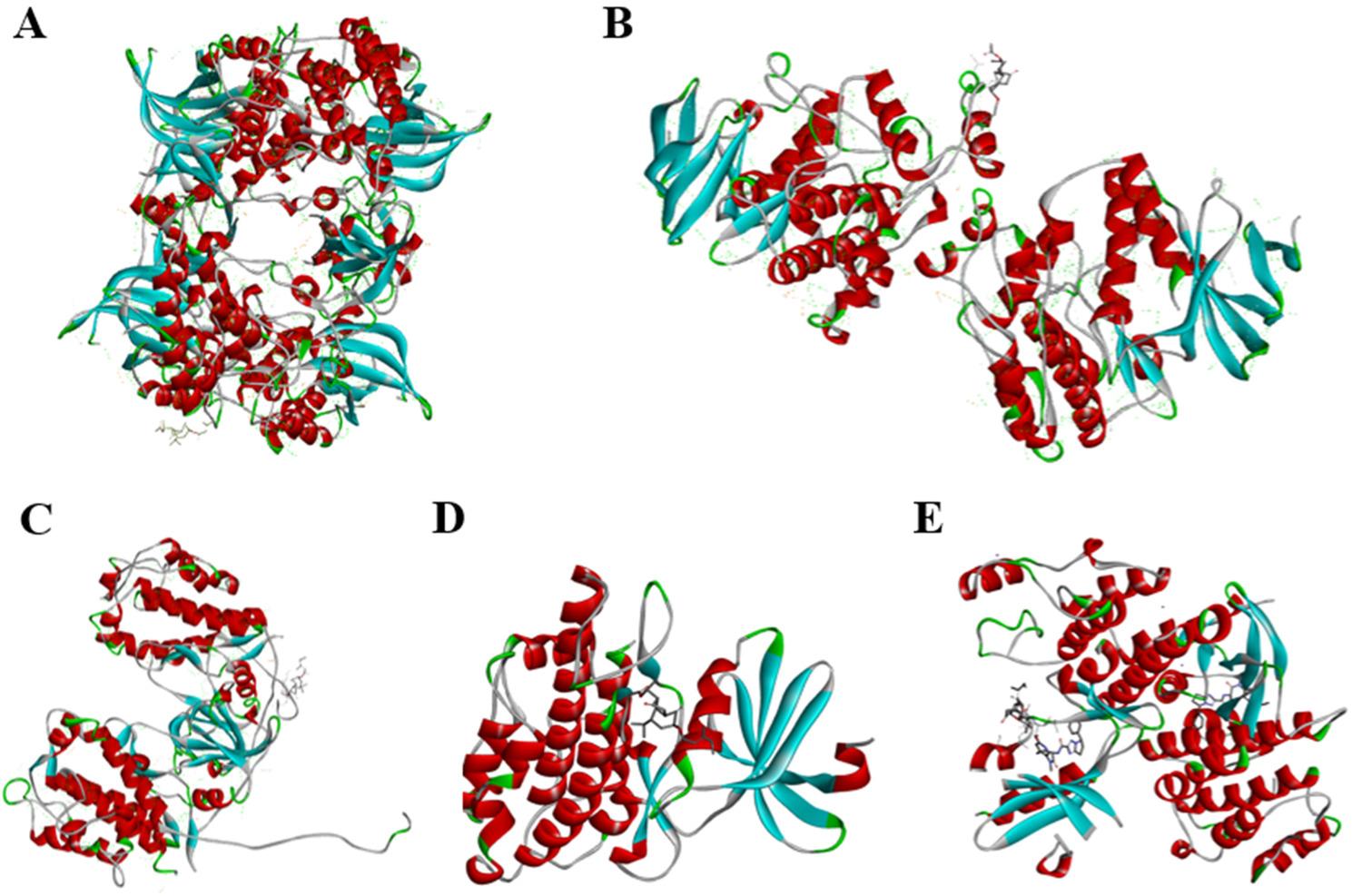
Identification of the top anti-inflammatory pathways associated with TS targets, including the MyD88, MAPK, and TLR4 signaling pathways. Key target genes identified through pathway analysis include* RIPK1*, *MAPKAPK2*, *MAPK9*, *MAPK11*, *MAP2K1*, *CDK1*, *RPS6KA3*, and *TBK1*, ranked by degree of interaction. Molecular docking analysis revealed the binding energy of TS with several key targets, indicating their potential as therapeutic sites: RIPK1: Binding energy of -31.0 kJ/mol, indicating the strongest affinity. RPS6KA3: Binding energy of -28.9 kJ/mol. MAPK11: Binding energy of -28.0 kJ/mol. MAPKAPK2: Binding energy of -26.4 kJ/mol. CDK1: Binding energy of -24.3 kJ/mol. Specific Docking Results: **A** TS and CDK1. **B** TS and MAPK11. **C** TS and MAPKAPK2. **D** TS and RPS6KA3. **E **TS and RIPK1

Molecular docking analysis revealed that the targets with the lowest binding energies to TS were RIPK1 (-31.0 kJ/mol), RPS6KA3 (-28.9 kJ/mol), MAPK11 (-28.0 kJ/mol), MAPKAPK2 (-26.4 kJ/mol), and CDK1 (-24.3 kJ/mol) (Fig. 5). Among these, the strongest affinity was observed between TS and RIPK1, indicating that TS may exert its anti-inflammatory effects by targeting RIPK1. These findings indicate that RIPK1 is a potential molecular target for TS in the regulation of sepsis-associated lung injury.

To further examine the expression levels of the target protein RIPK1 in the lung tissues of mice with sepsis-related lung injury, MLE12 cells were pretreated with TS for 1 h and subsequently stimulated with LPS for 8 h. Cellular proteins were then harvested, and the expression of RIPK1 was assessed using WB. The results demonstrated that TS intervention reduced RIPK1 expression in alveolar epithelial cells under LPS stimulation.

**Fig. 6:**
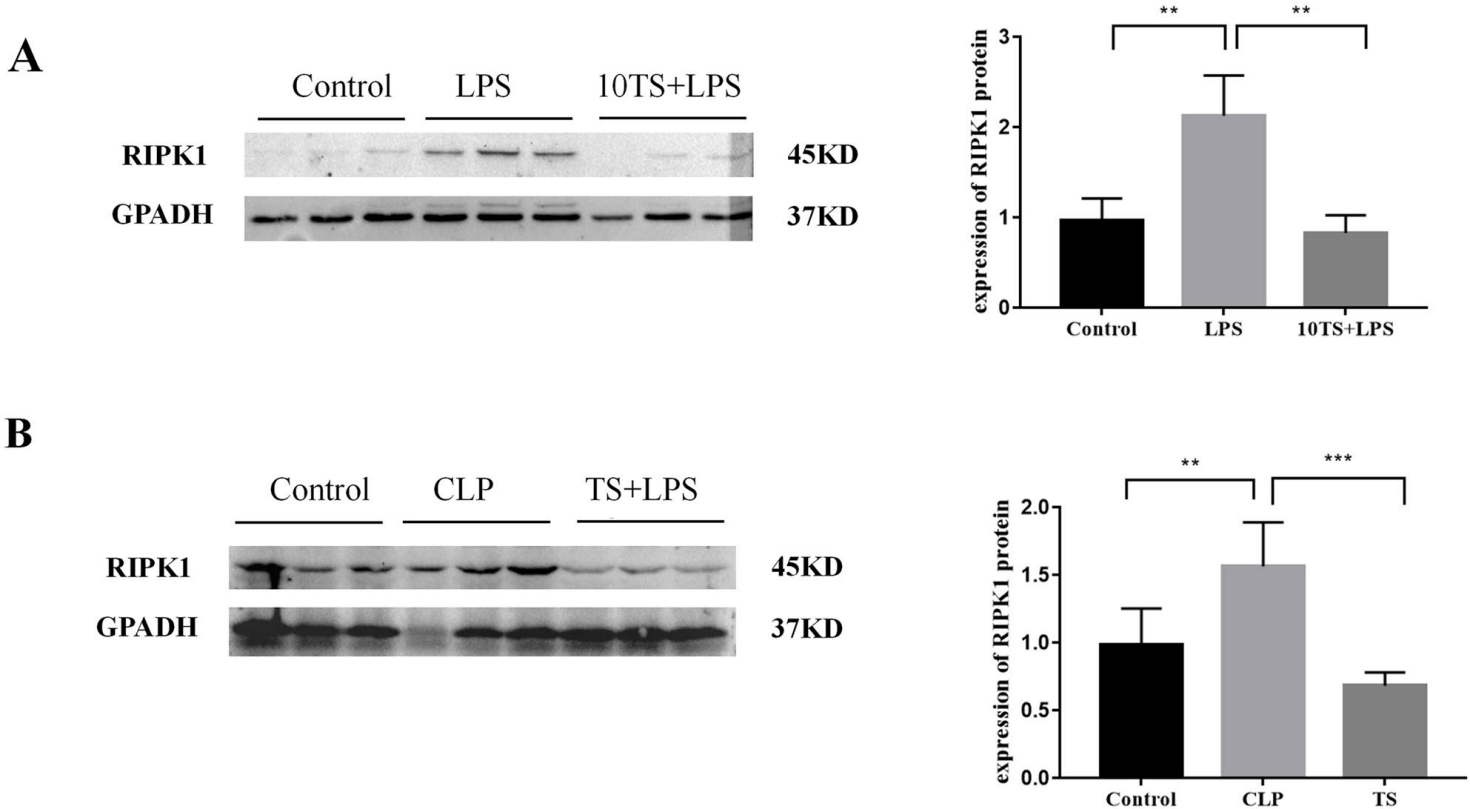
Expression of RIPK1 in TS-induced sepsis-associated lung injury. In vitro study: MLE12 cells were pretreated with TS for 1 h and then stimulated with LPS for 8 h. In vivo study: TS (30 mg/kg) was administered via oral gavage to CLP model mice 1 h before modeling, and lung tissue samples were collected 24 h post-modeling. **A** RIPK1 protein levels in MLE12 cells: Western blot analysis revealed that RIPK1 expression was significantly increased following LPS stimulation. However, TS intervention reduced RIPK1 protein levels compared to the LPS group. **B** RIPK1 protein levels in lung tissues of mice: In the CLP group, RIPK1 protein levels were elevated after modeling. TS intervention significantly reduced RIPK1 expression in lung tissues compared to the untreated CLP group. **P < 0.01, ***P < 0.001

Additionally, TS (30 mg/kg) was administered via gavage to CLP model mice one hour before modeling. Lung tissue samples were collected 24 h post-modeling, and WB was used to measure RIPK1 expression in the lung tissue. The findings indicated a significant reduction in RIPK1 expression in CLP mice treated with TS compared to untreated controls. These results collectively indicate that RIPK1 expression is upregulated in the lung tissues of mice with sepsis-related lung injury and in the inflammatory model of mouse lung epithelial cells, while TS intervention effectively downregulated RIPK1 expression (Fig. 6).

**Fig. 7:**
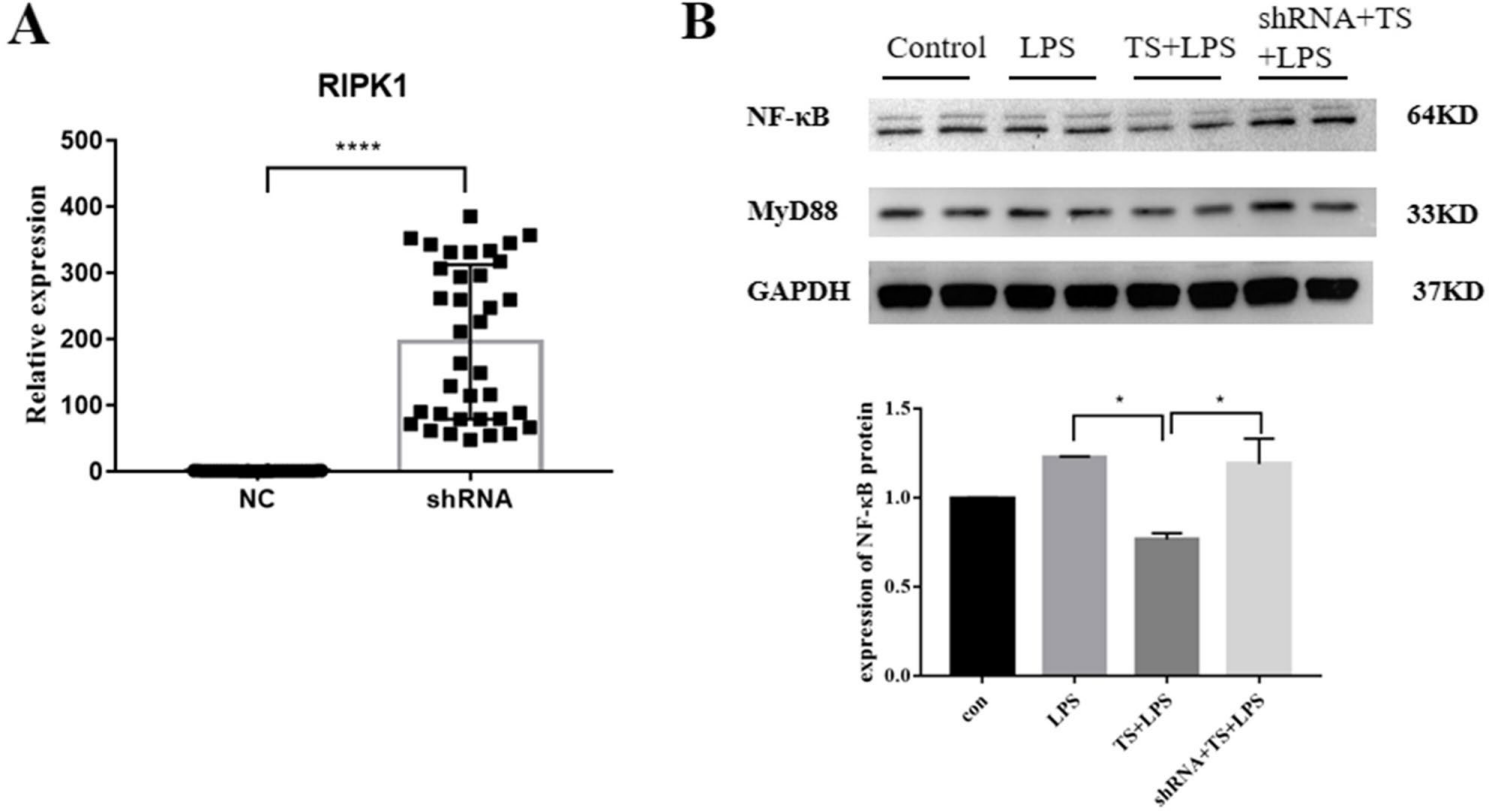
Changes in the expression of inflammatory pathway-related proteins in lung epithelial cells treated with TS after RIPK1 overexpression. MLE12 cells were transfected with a RIPK1 overexpression plasmid to assess the effect of TS on inflammatory pathway-related proteins under conditions of enhanced RIPK1 expression. Cells were pretreated with TS for 1 h, followed by stimulation with LPS for 8 h. Cellular proteins were then collected for analysis. **A** Transfection efficiency of *RIPK1*: qRT-PCR confirmed successful transfection of *RIPK1* into MLE cells, with a significant increase in *RIPK1* expression. **B **Protein levels of NF-κB p65:and MyD88: Western blot analysis revealed the following: In the LPS-treated group, the levels of NF-κB p65 and MyD88 proteins were significantly elevated. TS treatment reduced the expression of NF-κB p65 and MyD88 proteins compared to the LPS-treated group. However, in cells with RIPK1 overexpression, TS treatment had no significant effect on the levels of NF-κB p65 and MyD88, indicating that overexpression of RIPK1 negates the anti-inflammatory effects of TS. Statistical analysis revealed significant differences. *P < 0.05, ****P < 0.0001

To further assess the role of RIPK1, MLE12 cells were transfected with a RIPK1-overexpressing plasmid. Following successful transfection, the cells were pretreated with TS for 1 h and subsequently stimulated with LPS for 8 h. Cellular proteins were then harvested for analysis. It was observed that TS treatment decreased the expression levels of NF-κB p65 and MyD88 proteins in MLE12 cells compared to the LPS-treated group. However, TS treatment had no significant effect on NF-κB p65 and MyD88 levels in cells overexpressing RIPK1 (Fig. 7).

To further validate the role of RIPK1 in the inflammatory response associated with CLP-induced septic lung injury in juvenile mice, C57BL/6 mice aged 4 to 5 weeks were used. RIPK1 expression was inhibited through intraperitoneal injection of Necrostatin-1 (Nec-1) at a dose of 1.65 mg/kg for four weeks, while a saline injection was used as a control. Additionally, TS (30 mg/kg) was administered via oral gavage to the CLP model mice one hour before modeling. Lung tissue samples were collected 24 h after modeling, and WB confirmed significant inhibition of RIPK1 expression in lung tissues compared to the control group.

**Fig. 8:**
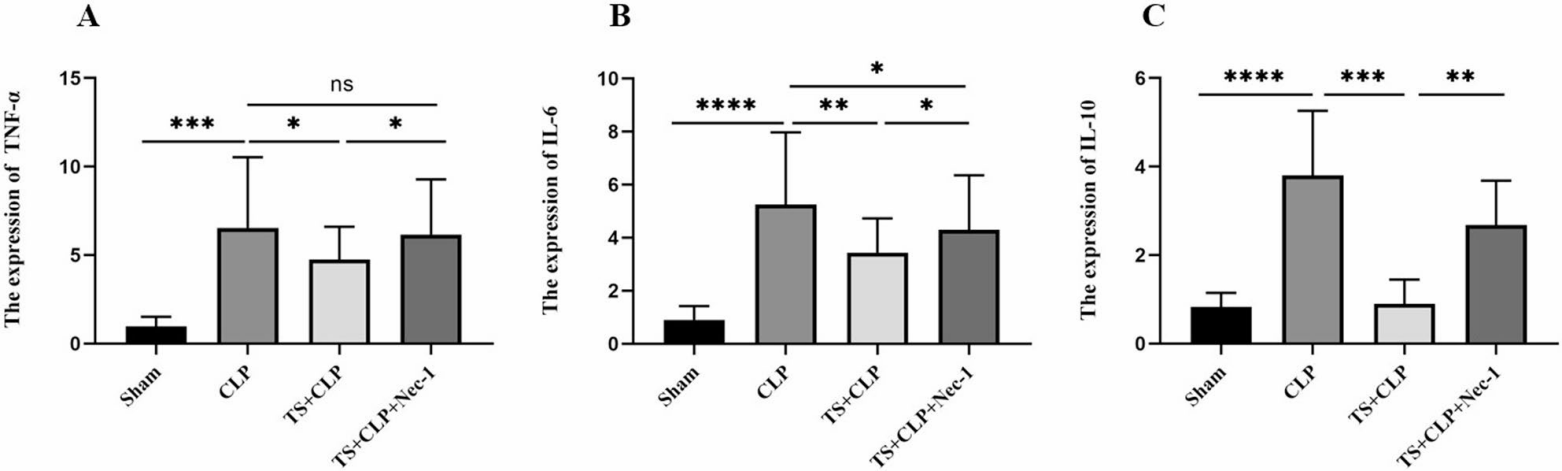
Experimental design for the evaluation of TS and Nec-1 in a sepsis-induced lung injury model. C57BL/6 mice aged 4–5 weeks were randomly assigned to four groups: Sham, CLP, TS + CLP, and TS + CLP+Nec-1, with 15 mice in each group. Nec-1 was administered intraperitoneally at a dose of 1.65 mg/kg daily for four weeks, while saline served as a control. TS (30 mg/kg) was given via oral gavage one hour before modeling in the TS + CLP and TS + CLP+Nec-1 groups. Lung tissue samples were collected 24 h after modeling. qRT-PCR was used to assess the expression levels of Cytokine expression. The results from Fig. 8 indicated successful transfection and expression of TNF-α, IL-6, and IL-10 in lung tissue. It is evident from the three images, labeled **A**, **B**, and **C**: In the CLP group, TNF-α, IL-6, and IL-10 expression levels were significantly elevated compared to the Sham group. TS treatment in the TS + CLP group significantly reduced the expression levels of TNF-α, IL-6, and IL-10 compared to the CLP group. *P < 0.05, **P < 0.01, ***P < 0.001,*** *P < 0.0001

The expression levels of inflammatory cytokines TNF-α, IL-6, and IL-10 were evaluated in Nec-1-treated CLP mice after TS intervention. These results were compared with those from the TS intervention group and the CLP group. Compared to the LPS group, the combined TS + Nec-1 + CLP treatment group exhibited significantly lower levels of inflammatory cytokines (Fig. 8). Furthermore, TS intervention alone reduced the levels of TNF-α, IL-6, and IL-10 in response to RIPK1 inhibition. 

These findings indicate that TS may modulate septic lung injury through targeted regulation of *RIPK1*.

## Discussion

Due to its high complexity and individual heterogeneity, the pathogenesis of sepsis remains a critical focus of ongoing research [23]. The inflammatory response plays a key role in sepsis and persists throughout the course of the disease. The primary signaling pathways mediating the inflammatory response in sepsis can be categorized into those associated with TLRs and non-TLR-related pathways. Among the TLR-associated pathways are the MyD88-dependent pathway, TRIF-dependent pathway, and the NOD-like receptor protein 3 (NLRP3)/caspase-1 signaling pathway [24]. 

The mitogen-activated protein kinase (MAPK) signaling pathway is a classical downstream pathway of the MyD88 signaling cascade. Activation of the TLR4/MyD88/p38MAPK pathway, triggered by pathogen-associated molecular patterns, facilitates signal transduction from the cell membrane to the nucleus. This process enhances NF-κB gene transcription, increasing NF-κB levels in the cytoplasm. Additionally, in the NLRP3/caspase-1 signaling pathway, activation of the NLRP3 inflammasome requires involvement of the TLR4/NF-κB signaling pathway.

NF-κB functions as a central downstream molecule within multiple signal transduction pathways, making its inhibition a critical therapeutic target in the treatment of sepsis.

In LPS-induced RAW264.7 cells and mouse peritoneal macrophages, TS suppresses the production of inflammatory mediators such as nitric oxide, TNF-α, and prostaglandin E2 [25]. In LPS-stimulated dendritic cells, TS exerts its anti-inflammatory effects by inhibiting the activation of NF-κB p65, MAPK, and IRF-3 signaling pathways [26]. 

Furthermore, TS promotes the interaction of the hypoxia-inducible factor 1-α/prolyl hydroxylase 2 (HIF-1α/PHD2) complex, which restores the elevated HIF-1α protein levels induced by PM2.5 exposure. This mechanism also inhibits NF-κB activation, effectively reducing the expression of inflammatory factors and mitigating PM2.5-induced damage to alveolar epithelial cells [27]. Additionally, TS suppresses the activation of MAPKs and NF-κB, thereby inhibiting the expression of inflammatory mediators and cytokines. This effect reduces macrophage inflammation induced by LPS and improves survival rates in mice with sepsis [11]. 

These findings indicate that TS reduces inflammation through multiple pathways. However, there have been no comprehensive studies that have systematically explored TS targets or its mechanisms in septic lung injury.

We employed both cellular and animal models and, through PCR, ELISA, and histopathological examination, found that TS can alleviate the inflammatory response in sepsis. This result prompted us to investigate the mechanism by which TS reduces sepsis-induced inflammation. The Traditional Chinese Medicine Systems Pharmacology Database and Analysis Platform and Swiss Target Prediction served as useful tools to identify potential targets of TS. Target-related genes were retrieved using the UniProt database. These targets were then uploaded to STRING to obtain protein-protein interaction networks of key proteins. Subsequently, the key targets were imported into the DAVID database (http://david.ncifcrf.gov) for GO functional and KEGG pathway enrichment analyses to predict the anti-inflammatory mechanism of TS, with the MYD88 signaling pathway implicated in this process.Using the RCSB PDB database, Discovery Studio 4.5 Client software, the PubChem database, PyRx software, and Vina software for a series of processing steps, we found that the binding energy between TS and RIPK1 was negative, indicating that TS can spontaneously bind to RIPK1. Therefore, we propose that RIPK1 may be a target through which TS alleviates sepsis-induced inflammation. Further experiments showed that inhibiting RIPK1 expression reduced the anti-inflammatory effect of TS, supporting RIPK1 as a functional target of TS in mitigating sepsis-related inflammation. Our study may have some limitations; for instance, overexpression of RIPK1 could provide more compelling bidirectional verification of these findings.

The results of this study demonstrate that TS exhibits a protective effect against sepsis-associated lung injury by significantly mitigating the severity of lung damage and reducing the release of inflammatory cytokines. *RIPK1*, a critical component of the NF-κB signaling pathway, serves as an important target for the protective action of TS, providing novel insights into potential therapeutic approaches for septic lung injury. In MLE-12 cells, the loss of RIPK1 reduces the secretion of pro-inflammatory cytokines, including IL-1α and IL-1β. In a C57BL/6 mouse model of sepsis, TS administration was associated with reduced lung injury and improved survival rates. Furthermore, the protective effect of TS was enhanced in the absence of RIPK1, highlighting that TS alleviates sepsis-induced ALI in mice through the inhibition of *RIPK1* expression.

Although TS has demonstrated anti-inflammatory properties in conditions such as sepsis, inflammatory bowel disease, leukemia, and asthma, studies on its mechanisms remain incomplete [22, 25]. Although TS consistently suppresses inflammatory responses, the pathways through which it acts vary across different diseases. Current research on the specific molecular pathways by which TS inhibits inflammation is limited, and further investigation is required to fully elucidate its mechanisms in diverse pathological contexts.

*RIPK1* is a member of the serine/threonine kinase family and plays a key role in innate immune signaling and cellular stress pathways [28]. Upon activation of TNFR1, a signaling complex known as complex I is assembled, which includes RIPK1, adaptor proteins such as TRADD (TNF receptor-associated death domain) and TRAF2 (TNF receptor-associated factor 2), E3 ubiquitin ligases, and inhibitors of apoptosis located on the cytoplasmic tail of the receptor [29]. 

Through the processes of reciprocal phosphorylation and ubiquitination of RIPK1, cellular survival and death pathways are tightly regulated. When RIPK1 undergoes ubiquitination, it forms a stable scaffold with IKKα/IKKβ (IκB kinase α/IκB kinase β), leading to the degradation of IκBα and the subsequent release and activation of NF-κB [30]. This balance in RIPK1 signaling is essential for managing cellular survival, programmed cell death, and inflammatory responses during inflammatory challenges [31]. However, the precise mechanisms by which *RIPK1* regulates these pathways remain incompletely understood, warranting further investigation.

RIPK1 protein was identified as a key target through network pharmacology and bioinformatics predictions based on the molecular and structural formulas of tussilagone. This finding was supported by extensive validation using both cellular and animal experiments. The results demonstrated that tussilagone mitigates the inflammatory response and alleviates septic lung injury by inhibiting the activity of RIPK1 protein.

Sepsis is an acute stress condition triggered by infection, characterized by the involvement of multiple mechanisms, including immune and metabolic dysregulation. Immune imbalance is a critical factor contributing to the excessive production of inflammatory cytokines observed in sepsis. Several studies have demonstrated that RIPK1 antagonists, such as Nec-1, effectively mitigate sepsis by blocking TNF-induced inflammatory responses [32]. These effects are mediated through the reduction of pro-inflammatory cytokines, decreased vascular and intestinal permeability, and inhibition of the coagulation cascade in vascular endothelial cells. In neonatal sepsis models, Nec-1 attenuates systemic and pulmonary inflammation through RIPK1 inhibition [33]. Similarly, in piglet sepsis models, Nec-1 pretreatment reduced jejunal injury and improved digestive and barrier functions, highlighting its protective effects against intestinal injury in sepsis [34]. Additionally, RIPK1 has been implicated in enhancing the production of granulocyte-macrophage colony-stimulating factor, IL-6, IL-8, and TNF under LPS stimulation, indicating its adverse role in septic pathology [35]. 

TS has been identified as having a protective effect against sepsis-related lung injury, with *RIPK1* serving as a key therapeutic target. This study is the first to establish a relationship among TS, *RIPK1*, and septic lung injury. TS exerted its protective effects primarily by modulating inflammatory cytokines, key inflammatory pathway proteins, and the expression levels of *RIPK1*. However, the precise mechanisms underlying these effects were not comprehensively explored, representing a limitation of the study. Specifically, the absence of in vivo *RIPK1* overexpression or knockout models limits our ability to conclusively establish whether the protective effects of TS are strictly dependent on RIPK1 at the organism level. Second, to validate the role of RIPK1, we relied on Nec-1, a pharmacological inhibitor widely used in necroptosis research. However, Nec-1 is known to exhibit off-target effects. Consequently, the observed protective effects attributed to RIPK1 inhibition may be partially confounded by Nec-1-mediated non-RIPK1 pathways.

Future studies employing more specific approaches—such as RIPK1 kinase-dead knock-in mice or next-generation selective RIPK1 inhibitors—are necessary to confirm the target specificity of TS. Furthermore, future research should focus on using small molecule probe technology to precisely target *RIPK1*, identify molecular binding sites between TS and *RIPK1*, and screen for safer and more effective therapeutic components. These efforts aim to enhance the safety and efficacy of drug therapies for sepsis-related conditions. Beyond validating this mechanism, exploring novel pathways by which TS protects against septic lung injury may provide innovative directions for the clinical management of septic lung injury.

## Conclusion

Tussilagone improves the survival rate of CLP mice and alleviates sepsis-induced lung injury and inflammatory responses in CLP mice. Through network pharmacology prediction and pharmacological inhibition of RIPK1 expression at the animal level, it was found that tussilagone alleviates the inflammatory response of sepsis-induced lung injury in CLP mice mediated by the MyD88/NF-κB pathway by acting on RIPK1. Meanwhile, tussilagone can reduce LPS-induced acute lung injury in MLE-12 cell sepsis models. Through network pharmacology prediction and RIPK1 overexpression at the cellular level, it was demonstrated that tussilagone mitigates LPS-induced cellular inflammatory responses mediated by the MyD88/NF-κB pathway by targeting RIPK1.

## Data Availability

All data generated or analysed during this study are included in this article. Further enquiries can be directed to the corresponding author.

## Abbreviations

TS: Tussilagone
LPS: Lipopolysaccharide
*RIPK1*: Receptor-interacting serine/threonine-protein kinase 1
RIP: Receptor interacting protein
ALI: Acute lung injury
TCM: Traditional Chinese medicine
MAPK: Mitogen-activated protein kinase
MyD88: Myeloid Differentiation Primary Response Protein 88
TLR4: TOLL Like receptor 4
TCMSP: Traditional Chinese Medicine Systems Pharmacology Database and Analysis Platform
GO: Gene Ontology
KEGG: Kyoto Encyclopedia of Genes and Genomes
CLP: Cecal ligation and perforation
SPF: Specific Pathogen Free
ELISA: Enzyme-Linked Immunosorbent Assay
DMSO: Dimethyl sulfoxide
HE: Hematoxylin and eosin
MLE12: Mouse lung epithelial cells
EP: Eppendorf
GAPDH: Glyceraldehyde -3-phosphate dehydrogenase
DMEM: Dulbecco's Modified Eagle Medium
qRT-PCR: Quantitative Real-Time PCR
TaKaRa: PrimeScript RT Master Mix
ANOVA: A one-way analysis of variance
NLRP3: NOD-like receptor protein 3
MAPK: Mitogen-activated protein kinase
TNF-α: Tumor necrosis factor-α
HIF-1α/PHD2: Hypoxia-inducible factor 1-α/prolyl hydroxylase 2
Nec-1: Necrostatin-1
PDB: Protein Data Bank
RCSB: Research Collaboratory for Structural Bioinformatics
PDBQT: Protein Data Bank, Partial Charge (Q), & Atom Type (T)
PBS: Phosphate buffer saline
